# Pure tone audiometry with and without specific ear protectors

**DOI:** 10.1016/S1808-8694(15)30577-2

**Published:** 2015-10-19

**Authors:** Carlos Antonio Rodrigues de Faria, Fabio Akira Suzuki

**Affiliations:** 1 Master's degree student in the Health Science graduate program at IAMSPE. Physician of the ENT unit, IAMSPE.; 2 Doctor in Medicine, UNIFESP-EPM. Master's degree in Otorhinolaryngology, UNIFESP-EPM. Coordinator of the graduate program at IAMSPE/ Vice-coordinator of the graduate program in otorhinolaryngology at IAMSPE. Hospital do Servidor Público Estadual “FMO”/ IAMSPE.

**Keywords:** audiometry, deafness

## Abstract

The authors evaluated pure tone audiometry with and without specific ear protectors.

**Aim:**

The purpose of this case control study was to measure the level of sound attenuation by earplugs.

**Materials and Methods:**

The evaluation included sixty ears of 30 subjects of both sexes, aged between 20 and 58 years, of various professional activities, with normal hearing thresholds, and following ten hours of auditory rest. The statistical results of pure tone audiometry at 500 to 4000 Hertz with and without specific ear protectors were analyzed.

**Results:**

These results were compared with those provided by the ear protector manufacturer.

**Conclusion:**

The results show that the rate of sound reduction was similar to the manufacturer's specifications.

## INTRODUCTION

Humankind has knows about the harmful effects of noise for 2500 years; there are reports of deafness in workers living close to the river Nile cataracts, in Ancient Egypt.^1^ For centuries, many investigators have written theses about the ill effects of noise on hearing. Only in the past 50 years, however, have hearing protection guidelines been edited, and worldwide concern with the effects of occupational and leisure-associated noise (discos, vehicle noise and individual noise) has led to further studies and control methods.^2^

The first studies relating certain professional activities with specific diseases were made 300 years ago. In 1700, the work “De Morbis Artificum Diatriba,” by the physician Bernardino Ramazzini, was published in Italy. It contained reports about diseases occurring in 50 professions that developed during the transformation of a predominantly agricultural society into an industrial one. Between 1760 and 1830, England hosted a movement that would forever change humankind; it was the Industrial Revolution, one of its consequences being severe health problems for the workers of that time. This situation led the British parliament to pass the Health and Morals of Apprentices Act in 1802, which is considered the first law for protecting workers.^3^

It is general knowledge that in the past few centuries humankind has generated an ever noisier society, which has made sound intensity into the most diffused form of pollution in the modern world and the most frequent of harmful agents against health within the work environment.^4^ According to the Occupational Safety and Health Administration (OSHA), about 1 million industry workers in the USA have noise-induced hearing loss, particularly at 1000, 2000, and 3000 Hz.^5^ North-American estimates show that 2.9 million workers are exposed to noise levels between 90 and 100 dBNA.^6^

In Brazil, the Ministry of Labor controls the safety and health conditions of workers through Regulating Acts that define harmful physical, chemical and biological agents against worker's health (NR-9). Unhealthy activities and operations are regulated by the NR-15; its annex 1 defines impact noise tolerance limits. These law, however, do not foresee periodic audiograms for workers exposed to chemical products, as decree 3080 of the Ministry of Social Security only recognizes the causal nexus for solvents.^7^

In 1994, the Comite Nacional de Ruido e Conservacao Auditiva do Brasil (Brazilian National Noise and Hearing Conservation Committee) defined the expression noise-induced hearing loss (NIHL) as the gradual loss of auditory acuity due to continuous exposure to elevated noise levels, always sensorineural, always irreversible, bilateral, rarely resulting in profound hearing loss, initiating at 3000, 4000 and 6000 Hz, and eventually affecting other frequencies. The victim of this cochlear condition present tinnitus and intolerance to loud noise.^8^ Pathophysiological noise-induced hearing alterations appear to be related to a decreased intracellular oxygen supply in Corti organ cells, leading to sensory epithelial edema and eventual loss of stereocilia, which occurs more rapidly during the first 10 to 15 years of exposure to noise.^9^, ^10^ Noise-induced injury is generally bilateral, insidious, progressive and irreversible, and is directly related to the duration of exposure and sound pressure levels.^8^ The NR-7 mandates audiometric tests only in individuals exposed to noise levels over 80 dBNA, which makes it difficult to have controls for assessing the auditory health of workers. Our occupational audiometries, therefore, lack an essential component, namely a control group of individuals exposed to lower intensity noise.^11^

NIHL is the second most common form of sensorial hearing loss, after presbyacusis (age-related hearing loss).^12^

According to Seligmam, an analysis of hearing loss inducing agents should include a clinical history, the occupational history, a physical examination, and an audiological evaluation. Pure tone audiometry, which is generally important for assessing hearing loss, provides few data about the communicating abilities of individuals with NIHL. This is relevant, since the difficulty in recognizing sentences is significant in these patients.^13^, ^14^

Certain agents may induce and accelerate hearing loss, particularly when associated with noise. These include: chemical products (mercury, cadmium, tobacco, lead, gold, arsenic and others), antibiotics (streptomycin, kanamycin, neomycin, chloranphenicol and others), diuretics (furosemide, etacrynic acid) and other substances (salicylates, quinine, nitrogen mustard).^15^

Two types of industrial noise that are harmful for hearing have been identified: impulse noise (firearms and metal banging against metal) and continuous noise, which is the commonest, and which causes more auditory damage, particularly at 4000 Hz.^16^

There are many studies about NIHL in various professions, such as symphonic orchestra musicians, as well as comparisons between Brazilian civil and military aviation professionals. Other studies have assessed household appliances as inducers of NIHL.^17^, ^18^, ^19^

It is known that hearing aids (sound amplification devices) may also injure auditory organs as an undesirable side effect.^20^ Ear protectors are a temporary solution for industrial noise; some workers in harmful environments refuse to use them routinely.^21^ Routine use of individual protection equipment (IPE) depends on the collaboration of employees themselves, on the ear protector model and on its adaptation to the external auditory canal; all of this means that actual auditory protection is lower than laboratory tests indicate.^22^

For purposes of assessing hearing loss, audiograms may be classified according to the five categories proposed by Freitas et al.:

a) losses over 25db at 4khz - 6khz - 8khz;
b) loss of 3khz;
c) loss of 2khz;
d) loss of 1khz;
e) loss of 500hz.^23^

Although these tests were done in normal hearing individuals, these parameters are useful for classifying hearing losses in the general population.

The diagnosis and treatment of NIHL is a major concern for otorhinolaryngologists, for public health reasons, for research and in daily medical office practice. The need to characterize hearing losses, particularly in industry workers, has shown that all of the conclusions and reports have been based on noise attenuation levels provided by IPE manufacturers that usually accompany their products. Some of these equipments do not state their attenuation levels, which required evaluating whether the available data were in fact compatible with the clinical evidence.

Two questions should be answered when assessing auditory injury due to professional or leisure activities: was the individual using ear protectors? If so, why was this equipment not effective in preserving hearing? A careful clinical history and supporting exams reveal important data about the progression of hearing loss, both of which are essential for the diagnosis and for medical reports. There are various ear protectors available in the market, such as earmuffs and earplugs (in-ear type). We decided to study noise attenuation levels of a specific earplug, thus encouraging our colleagues to investigate NIHL.

## OBJECTIVES

The purpose of this study was to compare noise attenuation data provided by IPE manufacturers, specifically about earplugs, with data obtained in our tests, thus assessing the actual noise attenuation for users in professional activities.

## MATERIAL AND METHOD

Audiometric testing (pure tone audiometry) was used in selecting 60 ears for this study - following 10 hours of auditory rest - between February and July 2003. The Research Ethics Committee approved this study (number 055/03).

### Inclusion criteria

Normal hearing adult individuals evaluated previously, with no race restrictions, having rested at least 10 hours before testing, and that authorized their participation in this study.

### Exclusion criteria

Individuals with any hearing loss of metabolic disease associated with deafness.

### Material

a Starkey mod. WRC audiometer (calibrated on 12/2002)
a Madsen Zodiac 901 immitance meter (calibrated on 12/2002)
an OtoSonic acoustic booth
circunaural earphone
earplug protector
Features: Pomp Plus C.A. 5745 - NRR 21dB- ANSI standard S 12.6 1984
NRR SF 17dB- ANSI standard S 12.6-1997
30 30 individuals aged between 20 and 58 years (22 female, 8 male).

### Audiometry criteria

Subjects underwent pure tone audiometry at all frequencies to exclude those with any hearing loss. Those that were selected were required to rest their hearing with ear protectors during 10 hours, after which a new audiometry was done. Results were collected at 500-1000-2000-4000 Hz and compared to the results obtained before ear protection. A statistical analysis was done at the end of testing.

Audiograms were classified according to OSHA standards to assess hearing loss.

### Statistical analysis

Two statistical analyses were done; the first (method A) was done based on the ANSI standards S 12.6 1984 (attenuation NR 21 dBNA) and the second analysis (method B) was done based on the ANSI standards S 12.6 1997 (attenuation NRsf 17 dBNA). These values are closer to those found in actual working environments.

Four z-tests were made (one for each frequency) to find whether ear protectors actually attenuated sound at the same intensity as the values indicated by manufacturers. This test was chosen because, as shown in Table 1 below, manufacturers provide expected noise attenuation values and a standard deviation.* Additionally, a descriptive analysis consisting of summarized measurements and box-plots** was made for the observed attenuations (in decibels) at each frequency. A t-test was done at the end to confirm the results.

**Table 1:** Expected noise attenuation values provided by manufacturers for each frequency, in Hertz

Frequency Hz	Attenuation (decibels)	Standard deviation
500	31,0	6,0
1000	32,0	5,2
2000	36,4	5,2
4000	37,6	6,9

### Method A

Attenuation NRR* 21 dBNA: ANSI standard S12.6 - 1984

### Method B

Attenuation NRRsf 17 dBNA: ANSI standards S12.6 -1997

Noise attenuation data obtained by method B (subjective method - actual ear method - placement by listeners) results in noise attenuation values closer to those observed in actual use within the working environment.

**Table 3:** Expected noise attenuation values provided by manufacturers for each frequency (in Hertz).

Frequency Hz	Attenuation (decibels)	Standard deviation
500	23,9	6,8
1000	23,5	5,9
2000	28,1	5,0
4000	29,2	6,9

## RESULTS

We used z-tests, one for each frequency; a complete statistical analysis was done in method A (NR 21 dBNA ANSI standard S 12.6 1984) and in method B (NRsf 17 dBNA ANSI standard S 12.6 1997). Noise attenuation data collected by method B are subjective, a real ear method in which listeners place the earmuffs themselves; in this cases, noise attenuation values are closer to those observed in actual working environments. The noise attenuation value in this method is the NRsf, which provides an estimate of noise in the protected ear after environmental noise is subtracted. NIOSH corrections (70% for plugs) should not be applied in this method, as attenuation values are close to actual values.

In method A (artificial ear), we obtained lower mean attenuation levels in 60 ears compared to those informed by manufacturers. At all frequencies, the manufacturer's variability was higher than observed results. A 35 dBNA attenuation was achieved only in one ear at 500 dBNA.

In method B, we obtained mean noise attenuation in 60 ears close to that informed by manufacturers, as shown on Table 2 at 500 and 1000 dBNA. The attenuation value was lower than expected at 2000; the value was higher than expected at 4000 dBNA.

**Table 2:** Summary of observed noise attenuation measurements (decibels) at each frequency.

Frequency Hz	Mean	Standard deviation	Median	Mode	Minimum	Maximum
500	22,8	3,8	25	20	15	35
1000	23,4	3,7	25	25	15	30
2000	27,3	4,5	27,5	30	20	35
4000	29,4	4,3	30	30	20	35

We undertook the t-test to confirm our results, and found that its result was different from that obtained in the z-test only at 500Hz; in this case, the standard deviation informed by the manufacturers is higher than that observed in the samples.^24^

## DISCUSSION

Noise may be defined as any undesirable auditory signal. Human ears respond differently from person to person to a given sound. It has been demonstrated that people subjected to noise over 85 dBNA may present hearing problems, annoyance, lack of concentration, insomnia and stress. Noise induced pathophysiological alterations in the ear appear to result from decreased intracellular oxygen levels in Corti organ cells, edema of the sensory epithelium and eventual loss of stereocilia.^9^, ^25^

It has also been demonstrated that noise may cause permanent hearing loss or temporary auditory fatigue; in both cases, audiograms show the acoustic notch effect, the so-called micro noise trauma.^26^

Only continuous audiometries provide information about the side, location and shape of the acoustic notch, which results from the influence of noise on auditory organs. After a day of work in noisy environments, workers present temporary hearing loss, from which they recover after a period of rest. This is an important sign for healthcare professionals, requiring them to increase the subject's awareness about the harmful effects of noise.^27^, ^28^

Table 2 shows that the mean noise attenuation in 60 ears at all frequencies was lower than the manufacturer's rating. Furthermore, we found that the variability informed by manufacturers was higher than the observed value.

**Chart 1:**
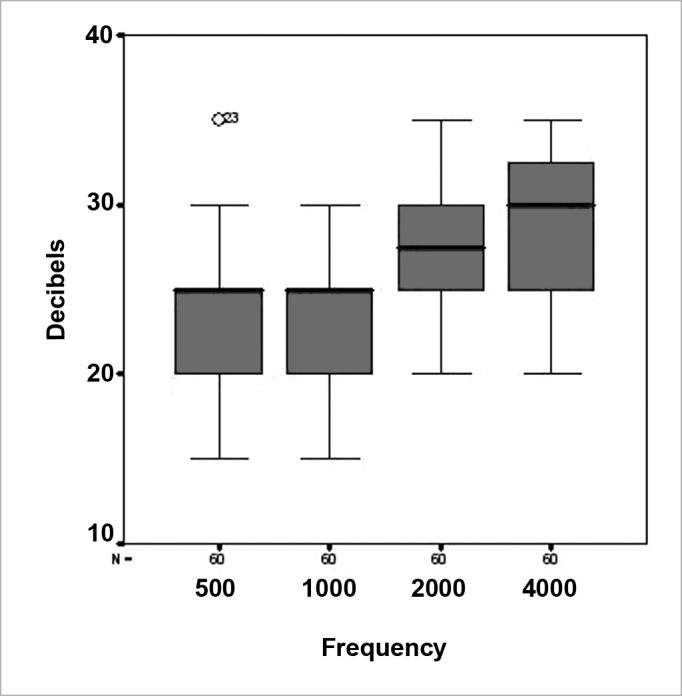
Box-plot comparing noise attenuation at 500, 100, 2000 and 4000 Hertz.

No ear achieved the expected attenuation, except at 500Hz. Maximum values at the other four frequencies were lower than the attenuation informed by manufacturers. Chart 1 shows that a 35 dBNA attenuation was present at 500Hz in only one ear (this was classified as an outlier); the second highest value was 30 dBNA, meaning that the expected attenuation was only achieved in one ear.

Table 2 shows that the most frequent attenuation value at any of the four frequencies was lower than expected values. As expected, the mean noise attenuation in 60 ears, at all frequencies, was close to that informed by manufacturers.

## CONCLUSION

The results shows that the earplug type protectors that we used in this study are effective in attenuating sound pressure levels in noisy environments. The information provided by manufacturers about noise attenuation levels were statistically comparable to those found in our study. The importance of ear protectors for public health reasons and in certain leisure activities was confirmed in this study. Medical reports may be based on these data, and companies should always provide protectors from manufacturers that provide noise attenuation data and its standard deviation.

Method A and B results (real ear) allow us to state that noise attenuation levels informed by manufacturers of the earplugs we used in this study were similar to the results. It is thus possible to use these data for calculating individual protection and as references in medical forensic reports.
